# The Role of Immature Granulocytes in Fibromyalgia: An Indicator of Subclinical Inflammation?

**DOI:** 10.3390/biomedicines13102511

**Published:** 2025-10-15

**Authors:** Mehmet Serhat Topaloğlu, Medeni Arpa, Bayram Şen, Hacer Bilgin Topaloğlu, Osman Cüre

**Affiliations:** 1 Department of Physical Medicine and Rehabilitation, Faculty of Medicine, Recep Tayyip Erdoğan University, Rize 53100, Turkey; 2 Department of Medical Biochemistry, Faculty of Medicine, Recep Tayyip Erdoğan University, Rize 53100, Turkey; medeni.arpa@erdogan.edu.tr; 3 Department of Medical Biochemistry, Recep Tayyip Erdoğan University Training and Research Hospital, Rize 53100, Turkey; 4 Department of Medical Biochemistry, Rize State Hospital, Rize 53100, Turkey; 5 Department of Rheumatology, Faculty of Medicine, Recep Tayyip Erdoğan University, Rize 53100, Turkey

**Keywords:** biomarker, fibromyalgia, inflammation, immature granulocyte

## Abstract

**Background:** Fibromyalgia (FM) is a chronic musculoskeletal disorder characterized by widespread body pain and various symptoms. Its etiology remains unclear to date. It has been associated with various pathogenic mechanisms, primarily inflammation. Immature granulocytes (IGs) have been proposed as a potential biomarker, playing a role in both inflammatory responses and prognosis in numerous diseases. No other study has investigated the count of immature granulocytes (IG#) and percentage of immature granulocytes (IG%) in FM patients. The aim of this study is to investigate the IG# and IG% in FM patients and to evaluate the relationship between these parameters and disease parameters. **Methods:** This retrospective observational study included 95 patients diagnosed with FM and 63 healthy control subjects matched for body mass index and gender. Biochemical, hematological parameters, erythrocyte sedimentation rate (ESR), C-reactive protein (CRP) and other inflammatory markers were recorded for both groups. Fibromyalgia Survey Diagnostic Criteria and Severity Scale (FSDC) and Fibromyalgia Impact Questionnaire (FIQ) scores were recorded for FM patients. **Results:** In FM patients, the IG% and IG# were significantly higher than in the healthy control group (*p* < 0.001, *p*: 0.006, respectively). There was no significant difference between the CRP and ESR values of the two groups. The platelet large cell count (PLCC) was significantly lower in the FM group (*p* < 0.032). **Conclusions:** IG levels can be used as a systemic early, sensitive, and low-cost marker in patients with FM. Based on our current knowledge, and considering the heterogeneous nature of FM, IG levels may serve as a meaningful tool in identifying subgroup elements reflecting an inflammatory phenotype. However, these findings require further validation through larger sample size, prospective studies, and advanced analyses including cytokine levels.

## 1. Introduction

Fibromyalgia (FM) is a chronic musculoskeletal disorder characterized by sleep disturbances, widespread body pain, difficulty performing daily activities, and various cognitive impairments. Although it is more common in women, it has been associated with many clinical conditions [1]. These include neuropsychiatric disorders, diabetes, infections and rheumatic diseases [2]. It is stated that the main reasons why FM is more common in women may be due to psychosocial predisposition, hormonal reasons, more frequent occurrence of autoimmune diseases, pain sensitivity and more pronounced response to pain [3,4,5]. The prevalence in the general population is around 1.3–8%. The exact cause of the disease is unknown [6]. Factors such as peripheral and central sensitization, inflammation, immunity, genetic factors, endocrinological causes, psychopathological factors and poor sleep quality are implicated in the etiology of FM [7]. Recent studies investigating the relationship between FM and systemic inflammation have suggested that proinflammatory cytokines, reactive oxygen species, and plasma-derived inflammatory factors may play a role in the pathogenicity of the disease [8,9].

The identification of new markers that can guide the diagnosis and follow-up of FM is clinically important. In addition to conventional blood count methods, more comprehensive hematological analyses, including immature granulocytes (IGs), can now be performed using flow cytometry. IGs are groups of cells belonging to the neutrophil series that are found in the bone marrow and have not reached full maturity (promyelocytes, myelocytes, metamyelocytes). Under normal physiological conditions, immature cells are not detected in the peripheral circulation, but as a result of various inflammatory cues, they can be released early from their reserve sites in the bone marrow and enter the circulation [10]. Studies have reported that IGs may increase in conditions such as sepsis and inflammatory rheumatic diseases and may be used as an important marker [11,12]. The role of these cells in FM and their response to inflammation is not clear.

Although several studies have investigated hematological indices in FM, to the best of our knowledge, no research has specifically examined the count and proportion of immature granulocytes. Our study is the first of its kind in this regard. Although the exact pathogenesis is not currently clarified, increasing evidence suggests that low-grade systemic inflammation and immune dysregulation play a role in the onset and persistence of FM. Recent studies have documented raised levels of proinflammatory cytokines and altered leukocyte profiles in FM patients, supporting the subtle inflammatory component hypothesis [8]. IGs, the early precursors of neutrophils, are known markers of bone marrow activation and subclinical inflammation. Unlike mature leukocyte subpopulations, IG appear in peripheral blood only in response to inflammatory stimuli or immune stress [13]. Therefore, evaluating IG count (IG#) and percentages (IG%) may provide additional information about the possible inflammatory mechanisms underlying FM. The main objective of our study is to determine the clinical significance of the number and IG% in peripheral blood as a biomarker of inflammatory activity in FM patients. Furthermore, this study will contribute to our knowledge base regarding the inflammatory underlying structure of FM and will guide future studies on this disease, the cause of which remains unknown today

## 2. Materials and Methods

### 2.1. Study Design and Participants

This retrospective study included a total of 158 female participants, comprising 95 patients with FM and 63 control subjects. Patients were diagnosed according to the 2016 criteria of the American College of Rheumatology (ACR). Individuals with inflammatory rheumatic diseases in addition to FM, those with autoimmune diseases, patients with psychiatric diagnoses, those with neurological diseases, cancer, kidney and liver failure, infectious diseases, cirrhosis, and major chronic conditions such as congestive heart failure, individuals under the age of 18 and over the age of 65, those with metabolic disorders, and those who smoke or drink alcohol, those receiving pharmacological or non-pharmacological treatment were excluded from the study. A healthy control group matched for gender was included in the FM patient group. The healthy control group was selected from healthy individuals who did not have chronic pain symptoms or any other illnesses and who visited the outpatient clinic with different complaints. Socio-demographic data, body mass index, age, and gender were recorded using a form. Hemogram parameters, C-reactive protein (CRP), erythrocyte sedimentation rate (ESR), and biochemistry parameters were recorded, and Fibromyalgia Survey Diagnostic Criteria and Severity Scale (FSDC) and Fibromyalgia Impact Questionnaire (FIQ) values were recorded.

### 2.2. Clinical Measurements

FSDC is a two-stage diagnostic classification system. In the first stage, the location of pain is determined. In the second stage, fatigue, waking up feeling unrested in the morning, cognitive symptoms, headache, abdominal pain/cramps, and the presence of depression are assessed. To diagnose FM, symptoms must have been present for at least 3 months, with a widespread pain index (WPI) ≥ 7, a symptom severity scale (SSS) ≥ 5 points, or a widespread pain index (WPI) of 4–6 and a symptom severity scale (SSS) ≥ 9 points. Additionally, a total score (FSDC) of ≥12 [14].

FİQ is an assessment tool consisting of 10 items that can be self-administered. The first section begins with physical activity and the other sections ask questions about weekly work loss, fatigue, pain, sleep disorders, well-being, morning stiffness, psychological state, anxiety, and general health status [15].

Hemogram parameters and IG# and IG% were measured using the Mindray BC-6200 (Mindray Bio-Medical Electronics Co., Ltd., Shenzhen, China) automated hematology analyzer, which classifies leukocytes based on multi-angle light scatter and fluorescence signals reflecting cell size, granularity, and nucleic acid content. Automated IG measurement offers rapid, reproducible, and operator-independent results compared with manual staining or microscopy. CRP test using the Beckman Coulter AU5800 (Beckman Coulter, CA, USA) automatic biochemistry analyzer, and Sedimentation Vision-C (YHLO Biotech Co., Ltd., Shenzhen, China) autoanalyzer.

Throughout the research process, all ethical principles were strictly adhered to, and ethical committee approval was obtained from the Non-Interventional Ethics Committee of Recep Tayyip Erdoğan University Faculty of Medicine (Number: E-40465587-050.01.04-1328. Decision No: 2025/19).

## 3. Statistics

All statistical analyses were performed using IBM SPSS Statistics for Windows, Version 29.0 (IBM Corp., Armonk, NY, USA). The distribution of continuous variables was assessed using the Kolmogorov–Smirnov test. Normally distributed variables were expressed as mean ± standard deviation (SD), while non-normally distributed variables were presented as median (minimum–maximum). Comparisons between the FM group and the healthy control group were conducted using the independent samples *t*-test or the Mann–Whitney U test, depending on the distribution of the variables. Since a significant difference in age was observed between the groups, analysis of covariance (ANCOVA) was employed for certain variables, with age included as a covariate, to obtain age-adjusted *p*-values. Correlations between biochemical parameters and disease severity scores were analyzed using Spearman’s rank correlation, adjusted for age. Correlation coefficients (rho) and their corresponding *p*-values were reported. To evaluate the diagnostic performance of specific biomarkers in predicting group membership (patient vs. control), receiver operating characteristic (ROC) curve analysis was performed. For each variable, the area under the curve (AUC) was calculated, and sensitivity, specificity, and the optimal cutoff value were determined using the Youden index. A *p*-value of < 0.05 was considered statistically significant for all tests.

## 4. Results

The study included individuals diagnosed with FM and a healthy control group. The mean age of participants was significantly higher in the FM group (*p* = 0.009). The immature granulocyte IG% was significantly higher in the FM group compared to the control group (*p* = 0.015; age-adjusted *p* < 0.001). Neutrophil levels were significantly lower in the FM group (*p* = 0.012; adjusted *p* = 0.029). The neutrophil–lymphocyte ratio (NLR) was found to be significantly lower in the FM group (*p* = 0.039); however, after adjusting for age, the significance remained borderline (*p* = 0.054). The mean platelet volume (MPV) was found to be significantly lower in the FM group (*p* = 0.035); however, this significance disappeared after adjustment for age (*p* = 0.105). No significant differences were found between the groups in terms of other parameters such as CRP, ESR, white blood cell, lymphocytes, monocytes, eosinophils, basophils, and hemoglobin (*p* > 0.05). The data for the groups are presented in Table 1.

**Figure 1:**
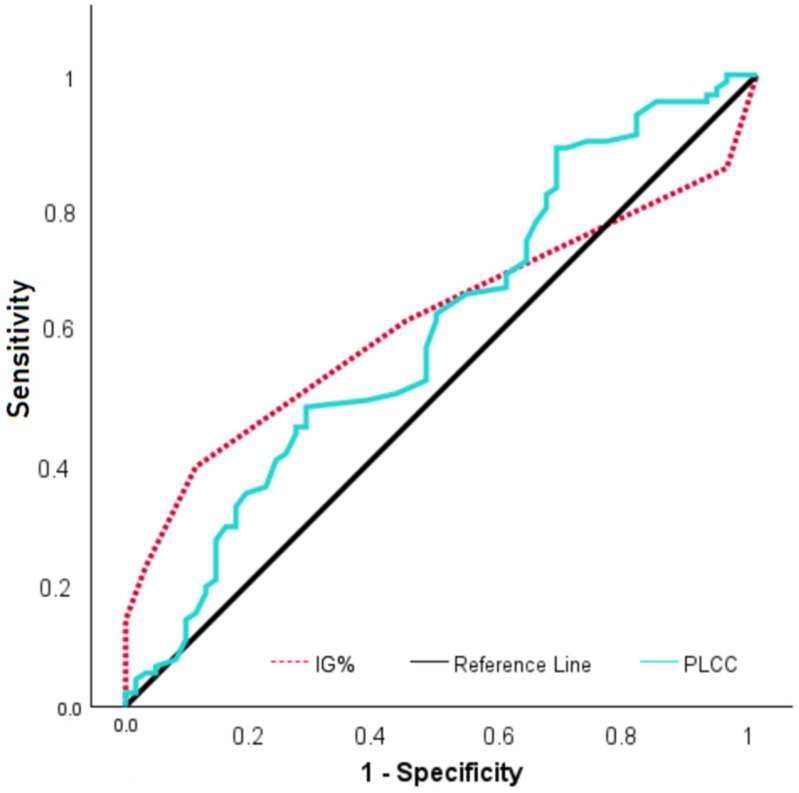
ROC curve of IG% and PLCC in FM patients.

ROC analysis was performed to evaluate the diagnostic performance of IG% and Platelet large cell count (PLCC) parameters for FM diagnosis. According to the results, when the IG% cut-off value was 0.25, sensitivity was 37.9% and specificity was 88.9% (AUC: 0.610; *p* = 0.013), while for PLCC with a cut-off value of 0.0103, sensitivity was 88.4% and specificity was 31.7% (AUC: 0.596; *p* = 0.039) (Figure 1). FM patients were dichotomized according to their IG% using a 0.25 cut-off. Functional outcomes were then compared between the groups accordingly. Although patients with IG < 0.25% showed slightly higher FIQ scores (71.79 ± 12.87) compared to patients with IG ≥ 0.25% (67.21 ± 14.81), this difference did not reach statistical significance (*p =* 0.115). Similarly, median FSDC scores were comparable between the two groups (19 [12–29] vs. 18 [14–30], *p =* 0.685).

## 5. Discussion

FM is a disease that seriously affects the overall quality of life, characterized by widespread musculoskeletal pain, bodily discomfort, cognitive abnormalities, also known as fibro fog, and sleep disturbances [16]. In addition to the inflammatory process in the central nervous system (CNS), peripheral inflammatory mechanisms are also reported to play a role in the development of the disease. The relationship between central and peripheral mechanisms is highly complex, with peripheral sensory inputs being modulated in the CNS to generate pain signals, and peripheral stimuli initiating central sensitization [17]. Activation of the central sensitization process leads to neurogenic inflammation, resulting in symptoms characteristic of FM, such as pain, peripheral swelling, and cognitive impairments [18,19]. In this context, some studies have highlighted that levels of proinflammatory cytokines such as IL-8, TNF-alpha, and IL-10 are significantly elevated in plasma and may play a role in the development of symptoms [9]. Genetic factors can indirectly affect hematological parameters by modulating the inflammatory response, influencing the neuroendocrine stress response, and altering cellular oxidative stress capacity. These changes may be associated with clinical symptoms in FM or may serve as biomarkers for assessing disease severity. FM can modulate pain sensitivity and immune response or cause changes in the immune response through epigenetic mechanisms such as DNA methylation under the influence of stress, inflammation, and environmental factors [20,21,22,23].

Studies conducted on dietary regimens in FM patients have indicated that vegan and vegetarian diet protocols are effective in suppressing systemic inflammation and improving disease symptoms as a result of their anti-inflammatory effect on the gut microbiome [24]. The anti-inflammatory effect of vegan and vegetarian diets is related to the type of food consumed. These diets provide high levels of fiber, vitamins, antioxidants, and minerals. The main anti-inflammatory substances contained in vegetarian diets are dietary fiber, salicylic acid, spices, and phytosterols. Phytosterols, in particular, are recognized as very powerful anti-inflammatory agents. It is thought that applying these diets to FM patients may contribute to the suppression of systemic inflammation [24]. Furthermore, a very recent study has established a causal relationship between FM and gut microbiota, and *Lactobacillus, Coprococcus 2*, and *Eggerthella* have been identified as strains that stand out in terms of risk [25]. The data presented in the literature suggest that both peripheral and central inflammation stages play a fundamental role in FM, and that chronic low-grade inflammation may influence symptoms by activating pain pathways

Hematological biomarkers are frequently used in the assessment of inflammation. There are conflicting findings regarding the diagnostic and prognostic values of parameters such as NLR, platelet/lymphocyte ratio (PLR) and MPV in FM. In the study by Aktürk and colleagues, CRP levels were found to be unchanged in FM patients, while NLR and MPV were found to be elevated. Farah and colleagues, however, reported no difference in NLR but elevated PLR and CRP levels [26,27]. Another study conducted with a more recent and broader patient group showed that NLR, monocyte/lymphocyte ratio (MLR) and PLR levels were similar in the FM and control groups, while sedimentation and CRP were higher in the FM group [28]. In our study, NLR, PLR, PDW, MPV, CRP, and sedimentation levels were similar between the FM and control groups, while IG# and IG% were higher in the FM group. This finding suggests that IG may also play a role in triggering the disease formation mechanism in FM through a silent inflammatory process. The inconsistency with the results of other studies may be due to differences in the demographic data of the participants included in the study. Furthermore, BMI was not assessed in some of these studies. This may have caused effects on CRP and other inflammatory markers.

It is noteworthy that NLR and PLR, which are markers of inflammation, were similar between groups in our study. These markers are more clinically significant in pathologies where the acute phase response is prominent and neutrophilic inflammation is predominant [11]. However, sensitivity may be limited in chronic and heterogeneous conditions where subclinical low-grade inflammation, such as FM, is prominent. This may explain why IG is more effective in demonstrating chronic inflammation. Numerous studies have linked IG levels to inflammatory and infectious processes. It has been reported that IG% levels can be used to monitor bacterial sepsis treatment and predict mortality [29]. In their study evaluating IG levels in patients with severe burns, Jeon and colleagues found that IG# were higher in patients without sepsis, while IG% was higher in patients with sepsis. They determined that IG% was significantly associated with the development of sepsis and that IG% was moderately useful in predicting the development of sepsis [30]. Korkmaz and colleagues found that it was useful in determining the severity of inflammation in the early stages of the disease in children with RSV bronchiolitis [31]. Aydın and colleagues reported that IG# and IG% were superior to other acute phase reactants in detecting inflammation, determining the prognosis and severity of the disease in patients with acute pancreatitis. In the same study, they found that NLR could be used as an indicator of inflammation but was not superior to other markers in predicting prognosis [32]. In a separate study, it was reported that IG% levels may be an important indicator in determining the severity and prognosis of severe pancreatitis [33].

Studies on the role of IG in inflammatory rheumatic diseases are increasing. Biyik and colleagues found high IG# and IG% in the Familial Mediterranean fever (FMF) group and suggested that IG may indicate subclinical inflammation and be used as a marker in disease follow-up [11]. In another study conducted by Akkeçeci and colleagues, it was suggested that IG# and IG% in patients with rheumatoid arthritis are associated with disease activity and can be used in disease activity monitoring [34]. In a single-center retrospective study conducted on patients with ankylosing spondylitis, it was stated that the rate and percentage of IG decreased after treatment and that it could be used as an important parameter in determining disease remission and treatment efficacy [35]. In our study, IG# and IG% were evaluated in FM patients for the first time in the literature; it was demonstrated that IG levels were significantly higher in the FM group. This finding provides new evidence regarding the inflammatory pathophysiology of FM and suggests that IG may be a potential biomarker reflecting subclinical inflammation. Meanwhile, it was found that IG# and IG% did not correlate with FIQ. This finding suggests that the clinical severity of the disease may not be directly related to inflammatory activity. In addition, although sedimentation and CRP levels did not show significant differences between groups, they showed a positive correlation with FIQ. Considering all results, we believe that the use of IG# and IG% would be useful in FM developing on a subclinical chronic inflammation background.

IGs show abnormal and sped up white blood cell production. The appearance of IGs in disease shows increased bone marrow activation, impaired maturation, or changes in cell production. Healthy individuals release only mature, differentiated cells into the circulating blood to ensure a stable and healthy immune response, whereas IGs are not found in the periphery [11,13]. However, in FM, an increase in early myeloid precursors, a decrease in bone marrow barrier function, and maturation problems in granulocytes may be observed in the peripheral circulation as a reactive response of the bone marrow to subclinical low-grade inflammation.

MPV has been associated with many diseases. MPV has been found to be high in FM patients, and high MPV values have been reported to contribute to an increased risk of cardiovascular disease [26,36]. Contrary to the findings here, Molina et al. found lower MPV values in FM patients, while Karlıbel et al. found MPV to be low in FM patients, similar to Molina et al., and did not detect any difference in platelet counts [37,38]. In our study, we found low MPV levels in FM patients, consistent with the findings, but when adjusted for age, this difference lost its significance. This difference between studies may be due to variability in clinical factors such as gender, age, and physical activity in patient selection.

We did not find any other study in the literature evaluating PLCC in patients with FM. In our study, PLCC was found to be significantly lower in the patient group. This result was also consistent with low MPV. However, neither of these two parameters was found to be associated with FIQ. In a study conducted by Jiang et al., platelet large cell ratio (PLCR) was found to be an independent predictor of thyroid malignancy [39]. In addition, alterations in platelet (PLT), PLCR, and MPV values have been observed in patients with schizophrenia, depression, and bipolar mania. Furthermore, a positive correlation between age and PLCR has been reported in the schizophrenia group [40]. The effects of low-grade systemic inflammation on the function and differentiation of hematopoietic stem cells (HSCs) are well known. Prolonged exposure to inflammatory signals can lead to the depletion and functional impairment of HSCs over time [41]. Due to these long-term inflammatory processes playing a role in the pathogenesis of FM, there may be a subclinical slowing in platelet formation and a corresponding slowing in the transition of young platelets to the peripheral blood in these patients, which may explain the low PLCC and PLCR values in our study. However, the cross-sectional nature of our study limits the generalizability of these findings. In this context, we believe that further studies with larger cohorts are needed to determine the role of PLCC and PLCR in the etiopathogenesis of FM.

This study has some limitations. The relatively small sample size represents a potential limitation, as it may constrain the generalizability of the study’s findings. In addition, owing to the retrospective design of the study, participants with incomplete data were excluded from the analysis. Proinflammatory cytokine levels were not measured in either the patient group or the control group; therefore, the inflammatory status of the participants could not be evaluated in detail. Another limitation of our study is that IG results were derived solely from an automated hematology analyzer without validation by flow cytometry or peripheral smear review, which may introduce method-dependent bias. Another potential limitation of this study is that IG# may increase not only in inflammatory conditions but also under physiological or psychological stress. Although FM is associated with chronic stress, all blood samples in this study were obtained under standardized conditions from outpatients, and individuals with active infection, autoimmune, or psychiatric disorders were excluded to minimize this effect. Therefore, while stress-related mechanisms might have contributed partially, the persistent elevation of IG parameters observed in FM patients suggests that a subclinical inflammatory process is also likely involved.

In conclusion, IG levels can be used as a systemic early, sensitive, and low-cost marker in patients with FM. Based on our current knowledge, considering the heterogeneous nature of FM, IG levels may serve as a meaningful tool in identifying subgroup elements reflecting an inflammatory phenotype. However, these findings require further validation through larger sample size, prospective studies, and advanced analyses including cytokine levels.

## Figures and Tables

**Table 1:** Comparison of clinical and hematological parameters in FM and control groups.

	FM	Healthy Control	*p* *	*p* **
	Mean ± SD; Median (Min-Max)	Mean ± SD; Median (Min-Max)		
Age	44.6 ± 9.4	40.37 ± 10.33	0.009	
FIQ	70.06 ± 13.74			
FSDC	20 ± 3.83			
ESR (mm/h)	9 (2–33)	10 (2–24)	0.562	0.364
CRP (mg/L)	2.37 (0.2–16)	1.8 (0.2–16.26)	0.537	0.803
IG%	0.2 (0–1.1)	0.1 (0–0.4)	0.015	<0.001
IG# (×10^3^/μL)	0.01 (0–0.09)	0.01 (0–0.03)	0.241	0.006
Albumin (g/L)	44.7 ± 2.5	45.3 ± 2.4	0.169	0.348
WBC (×10^3^/μL)	6.67 ± 1.46	7.17 ± 1.71	0.05	0.097
NEU (×10^3^/μL)	3.91 ± 1.09	4.39 ± 1.32	0.012	0.029
BA (×10^3^/μL)	0.03 (0.01–0.15)	0.03 (0.01–0.08)	0.695	0.988
EO (×10^3^/μL)	0.14 (0–0.53)	0.11 (0.01–0.53)	0.355	0.519
LYM (×10^3^/μL)	2.15 ± 0.63	2.15 ± 0.58	1.000	0.877
MON (×10^3^/μL)	0.39 (0.23–3.43)	0.44 (0.18–0.74)	0.176	0.997
RBC (×10^3^/μL)	4.51 (3.55–6.13)	4.38 (3.76–5.2)	0.125	0.274
HGB (g/dL)	13.26 ± 1.13	13.03 ± 0.96	0.185	0.341
HCT (%)	39.44 ± 3.14	38.83 ± 2.65	0.202	0.418
MCV (fL)	87.92 ± 4.33	88.21 ± 4.92	0.692	0.519
MCH (pg)	29.54 ± 1.69	29.59 ± 1.9	0.861	0.755
MCHC (g/dL)	33.59 ± 0.79	33.54 ± 0.79	0.702	0.533
PLT (×10^3^/μL)	278.37 ± 66.23	277.03 ± 71.95	0.905	0.94
MPV (fL)	10.45 ± 1.29	10.89 ± 1.22	0.035	0.105
PDW (%)	16 (10.4–16.9)	16 (15.5–16.8)	0.989	0.431
PCT (%)	0.29 ± 0.06	0.3 ± 0.07	0.244	0.269
NLR	1.83 (0.71–4.35)	2 (0.95–4.86)	0.039	0.054
BMI (kg/m^2^)	26.95 (20.57–40.26)	25.59 (19.72–48)	0.276	0.688
PLR	136.44 ± 40.43	134.72 ± 41.98	0.797	0.931
PLCC (×10^3^/μL)	77.13 ± 19.14	85.84 ± 24.96	0.014	0.032
PLCR (%)	29.04 ± 9.14	31.86 ± 8.43	0.038	0.077

*: Obtained from Independent Sample Tests; **: Adjusted for age. BMI: Body mass index, IG%: immature granulocyte percentage, IG#: immature granulocyte count, PLCR: platelet large cell ratio, PLCC: platelet large cell count; WBC: white blood cell; ESR: erythrocyte sedimentation rate; LYM: lymphocyte; MON: monocyte; NEU: neutrophil; BA: Basophil; EO: eosinophile; HGB: hemoglobin; PLT: platelet; NLR: neutrophil to lymphocyte ratio; PLR: platelet to lymphocyte ratio; MPV: mean platelet volume: MCV: mean corpuscular volume; RBC: Red Blood Cells; PDW: Platelet distribution width; MCHC: Mean Corpuscular Hemoglobin Concentration; MCH: mean corpuscular hemoglobin; CRP: C-reactive protein; FSDC: Fibromyalgia survey diagnostic criteria and severity scale; FİQ: fibromyalgia impact questionnaire.

## Data Availability

The datasets used and/or analyzed during the present study are available from the corresponding author upon reasonable request.
